# Mineral and bone disorder in Chinese dialysis patients: a multicenter study

**DOI:** 10.1186/1471-2369-13-116

**Published:** 2012-09-21

**Authors:** Xianglei Kong, Luxia Zhang, Ling Zhang, Nan Chen, Yong Gu, Xueqing Yu, Wenhu Liu, Jianghua Chen, Liren Peng, Weijie Yuan, Hua Wu, Wei Chen, Minhua Fan, Liqun He, Feng Ding, Xiangmei Chen, Zuying Xiong, Jinyuan Zhang, Qiang Jia, Wei Shi, Changying Xing, Xiaoling Tang, Fanfan Hou, Guiyang Shu, Changlin Mei, Li Wang, Dongmei Xu, Zhaohui Ni, Li Zuo, Mei Wang, Haiyan Wang

**Affiliations:** 1 Renal Division, Department of Medicine, Peking University First Hospital; Peking University Institute of Nephrology; Key Laboratory of Renal Disease, Ministry of Health of China; Key Laboratory of Chronic Kidney Disease Prevention and Treatment (Peking University), Ministry of Education, Beijing, China; 2 Department of Nephrology, China-Japan Friendship Hospital, Beijing, China; 3 Department of Nephrology, Ruijin Hospital, Shanghai Jiao Tong University, School of Medicine, Shanghai, China; 4 Renal Division, Xinhua Hospital, Shanghai Jiao Tong University, School of Medicine, Shanghai, China; 5 Department of Nephrology, the First Affiliated Hospital, Sun Yat-sen University, Guangzhou, China; 6 Department of Nephrology, Beijing Friendship Hospital, Capital Medical University, Beijing, China; 7 Kidney Diseases Center, First Affiliated Hospital, Zhejiang University School of Medicine, Hangzhou, China; 8 Department of Nephrology, Beijing Chaoyang Hospital, Capital Medical University, Beijing, China; 9 Department of Nephrology, Shanghai First People s Hospital, Shanghai Jiaotong University, Shanghai, China; 10 Department of Nephrology, Beijing Hospital, Beijing, China; 11 Department of Nephrology, Xijing Hospital, Fourth Military Medical University, Xi’an, China; 12 Department of Nephrology, Peking University Third Hospital, Beijing, China; 13 Department of Nephrology, Shuguang Hospital, Shanghai University of Traditional Chinese Medicine, Shanghai, China; 14 Department of Nephrology, Huashan Hospital, Fudan University, Shanghai, China; 15 Department of Nephrology, The General Hospital of the People's Liberation Army, Beijing, China; 16 Renal Division, Hospital Peking of Shenzhen, Shenzhen, China; 17 Department of Nephrology, The 455th Hospital of PLA, Shanghai, China; 18 Department of Nephrology, Xuanwu Hospital, Capital Medical University, Beijing, China; 19 Department of Nephrology, Guangdong General Hospital, Guangzhou, China; 20 Renal Division, Jiangsu Provincial Hospital, Nanjing, China; 21 Department of internal medicine, Shantou Central Hospital, Shantou, China; 22 Department of Nephrology, Nanfang Hospital, The Southern Medical University, Guangzhou, China; 23 Department of Nephrology, Fujian Provincial Hospital, Fuzhou, China; 24 Department of Nephrology, Changzheng Hospital, Second Military Medical University, Shanghai, China; 25 Department of Nephrology, Sichuan Provincial People’ s Hospital, Chengdu, China; 26 Department of Nephrology, Qianfoshan Hospital, Shandong University, Jinan, China; 27 Renal Division, Renji Hospital, Shanghai Jiao Tong University School of Medicine, Shanghai, China; 28 Department of Nephrology, Peking University People’s Hospital, Beijing, China; 29 Institute of Nephrology and Division of Nephrology, and Key Laboratory of Ministry of Health, Peking University First Hospital, 8 Xishiku Street, Xicheng District, Beijing, 100034, China

**Keywords:** End stage renal disease, Mineral and bone disorder, Epidemiology

## Abstract

**Background:**

Mineral and bone disorder (MBD) in patients with chronic kidney disease is associated with increased morbidity and mortality. Studies regarding the status of MBD treatment in developing countries, especially in Chinese dialysis patients are extremely limited.

**Methods:**

A cross-sectional study of 1711 haemodialysis (HD) patients and 363 peritoneal dialysis (PD) patients were enrolled. Parameters related to MBD, including serum phosphorus (P), calcium (Ca), intact parathyroid hormone (iPTH) were analyzed. The achievement of MBD targets was compared with the results from the Dialysis Outcomes and Practice Study (DOPPS) 3 and DOPPS 4. Factors associated with hyperphosphatemia were examined.

**Results:**

Total 2074 dialysis patients from 28 hospitals were involved in this study. Only 38.5%, 39.6% and 26.6% of them met the Kidney Disease Outcomes Quality Initiative (K/DOQI) defined targets for serum P, Ca and iPTH levels. Serum P and Ca levels were statistically higher (P < 0.05) in the HD patients compared with those of PD patients, which was (6.3 ± 2.1) mg/dL vs (5.7 ± 2.0) mg/dL and (9.3 ± 1.1) mg/dL vs (9.2 ± 1.1) mg/dL, respectively. Serum iPTH level were statistically higher in the PD patients compared with those of HD patients (P = 0.03). The percentage of patients reached the K/DOQI targets for P (37.6% vs 49.8% vs 54.5%, P < 0.01), Ca (38.6% vs 50.4% vs 56.0%, P < 0.01) and iPTH (26.5% vs 31.4% vs 32.1%, P < 0.01) were lower among HD patients, compared with the data from DOPPS 3 and DOPPS 4. The percentage of patients with serum phosphorus level above 5.5 mg/dL was 57.4% in HD patients and 47.4% in PD patients. Age, dialysis patterns and region of residency were independently associated with hyperphosphatemia.

**Conclusions:**

Status of MBD is sub-optimal among Chinese patients receiving dialysis. The issue of hyperphosphatemia is prominent and needs further attention.

## Background

Mineral and bone disorder (MBD) is a common complication of chronic kidney disease (CKD), and has been associated with increased risk of cardiovascular calcification, arterial dysfunction, morbidity and mortality [1,2]. Recently, increased attention has been focused on MBD, and hyperphosphatemia is a central role. A meta-analysis revealed that hyperphosphatemia is an independent risk factor for mortality among CKD patients [3]. Gradual decline in renal phosphorus clearance during the progression of CKD leads to an increase in serum phosphorus concentrations [4], which plays an important role in development of MBD [5]. Hyperphosphatemia may also contribute to vascular calcification and therefore higher risk of cardiovascular morbidity [1,2].

The Kidney Disease Outcomes Quality Initiative (K/DOQI) clinical practice guidelines for MBD were released in 2003, aiming at assisting nephrologists in developing an integrated approach to the diagnosis and management of MBD. Since then, studies have suggested an improvement in treatment of MBD in many countries [6,7]. However, studies regarding the status of MBD treatment in developing countries are extremely limited, where the accessibility to healthcare resources and structure of health care system are different. Therefore, the Practice Patterns and Improvement Study of Bone Metabolism and Disease in patients with CKD (PPIS) was initiated to provide national data on the status of MBD treatment among patients receiving dialysis in China.

## Methods

### Study population

**Figure 1:**
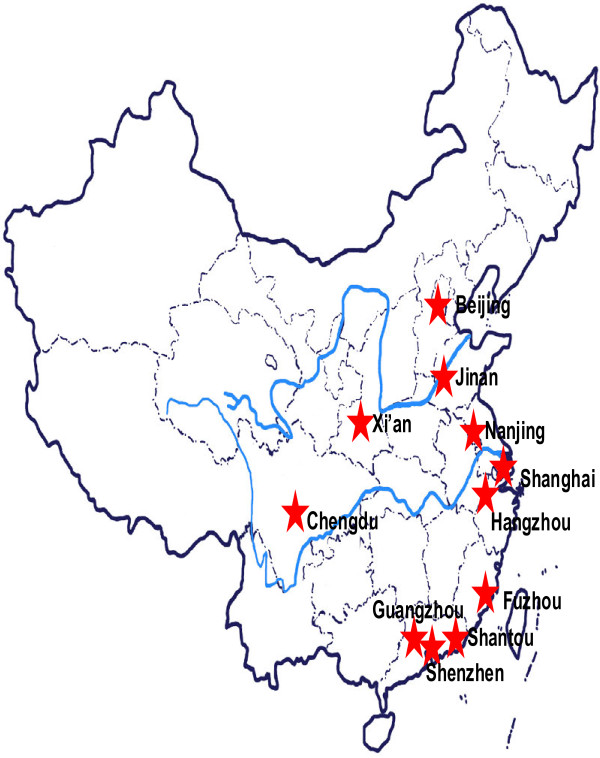
A map marked with study centers.

Twenty eight renal divisions from 9 provinces in China representing different geographic distribution and economic status were recruited as a convenience sample on a voluntary basis from January 2006 to June 2006. A map marked with study centers is provided in Figure 1. Adult ESRD patients receiving stable hemodialysis (HD) or peritoneal dialysis (PD) for at least 3 months were included in this study. Patients with renal tubule acidosis, multiple myeloma, idiopathic hypercalcemia or metastatic carcinoma of bone were excluded from the study. The ethics committee of Peking University First Hospital approved the study. All participants gave written informed consent prior to data collection.

### Data collection

A questionnaire documenting information of sociodemographic status, personal and family health history, dialysis prescription, and medication for MBD were finished by doctors or nurses. Then a blood sample were drawn after an overnight fast of at least 8 hours, and before the HD session for HD patients. Parameters related to MBD, including serum phosphorus, calcium and intact parathyroid hormone concentration (iPTH) were measured at central laboratory of each study center. Serum phosphorus and serum calcium were measured by spectrophotometry assay. iPTH was measured by immunoradiometric or immunochemiluminometric assays. Corrected calcium was calculated as total calcium (mg/dL) + 0.8 × (4 - serum albumin (g/dL)).

### Statistical analyses

Data were presented as proportions for categorical variables and mean ± standard deviation (SD) or median [interquartile range (IQR)] for continuous variables. Comparisons between HD and PD patients were made using *t* - test or Wilcoxon rank-sum test for continuous variables, and chi-square test for categorical variables.

According to the K/DOQI guideline [8], patients with corrected serum calcium from 8.4 to 9.5 mg/dL (2.10 to 2.37 mmol/L), phosphate from 3.5 to 5.5 mg/dL (1.13 to 1.78 mmol/L) and iPTH from 150 to 300 pg/mL (150 to 300 ng/L) were considered optimal. The percentage of participants within the optimal range and outside the optimal range (above or below) was compared with the results of Dialysis Outcomes and Practice Study (DOPPS) 3 (2007) and DOPPS 4 (2010) [9].

Factors associated with hyperphosphatemia were analyzed using Logistic regression models. Factors in the models included age (continuous), sex (female vs male), dialysis pattern (PD vs HD), serum albumin level (continuous), hemoglobin level (continuous), and region of residency (south vs. north).

All analyses were performed by SPSS statistical package, version 16.0 (SPSS, Inc., Chicago, IL). A P value of less than 0.05 is considered statistically significant.

## Results

Altogether 2074 patients were recruited, including 1711 HD patients and 363 PD patients. The average age was 57.5 ± 14.3 years, and 53.8% were males. The mean vintage of HD and PD were 47.8 ± 41.2 months and 24.1 ± 20.0 months, respectively. Compared with HD patients, PD patients were older, and had higher percentage of females. The main cause of ESRD was glomerulonephritis (47.5%), while the percentage of diabetic nephropathy was higher among PD patients compared with HD patients (22.0% vs 8.9%) (Table 1).

**Table 1:** Characteristics of the participants and comparison between HD and PD

**Variables**	**All participants (n = 2074)**	**HD (n = 1711)**	**PD (n = 363)**	**P value**
Age (years)	57.5 ± 14.3	57.1 ± 14.2	59.0 ± 14.4	0.03
Male (%)	53.8	55.8	44.1	< 0.001
Duration of dialysis (months)	33.6 ± 40.7	47.8 ± 41.2	24.1 ± 20.0	< 0.001
Cause of ESRD (%)				
Glomerulonephritis	47.5	49.4	38.6	< 0.001
Hypertension	12.1	11.6	14.6	0.11
Diabetes	11.2	8.9	22.0	< 0.001
Interstitial nephritis	7.4	7.6	6.3	0.44
Obstructive nephropathy	2.4	2.4	2.5	0.85
Others	19.4	20.1	16.0	0.18
Hemoglobin (g/L)	101.0 ± 2.1	101.1 ± 2.0	99.3 ± 2.1	0.14
Serum albumin (mg/L)	37.5 ± 5.0	37.8 ± 4.8	36.1 ± 5.7	< 0.001
Corrected calcium (mg/dL) (%)				
Mean ± SD	9.3 ± 1.1	9.3 ± 1.1	9.2 ± 1.1	0.03
< 8.4	19.4	19.4	19.4	1.00
8.4-9.5	39.6	38.6	44.0	0.07
> 9.5	41.0	42.0	36.6	0.06
Phosphorus binder prescription (%)	70.5	71.4	66.7	0.35
Vitamin D prescription (%)	61.2	64.6	43.2	< 0.001
Phosphorus (mg/dL) (%)				
Mean ± SD	6.2 ± 2.1	6.3 ± 2.1	5.7 ± 2.0	< 0.001
< 3.5	5.8	5.0	9.6	< 0.01
3.5-5.5	38.5	37.6	43.0	0.06
> 5.5	55.6	57.4	47.4	< 0.01
Phosphorus binder prescription (%)	73.0	72.6	75.0	0.65
Vitamin D prescription (%)	66.6	66.5	67.5	0.85
iPTH (pg/mL) (%)				
Median (IQR)	269.0 (132.0 - 472.9)	265.0 (127.2 – 456.3)	304.5 (162.0 – 486.7)	0.03
< 150	27.8	29.0	22.3	< 0.01
Vitamin D prescription (%)	48.1	52.3	23.5	< 0.001
150-300	26.6	26.5	27.3	0.74
Vitamin D prescription (%)	67.9	72.4	49.5	< 0.001
> 300	45.5	44.5	50.4	0.05
Vitamin D prescription (%)	78.2	77.5	81.2	0.34
Phosphorus binder prescription (%)	69.4	70.8	64.0	0.03
Vitamin D prescription (%)	67.4	69.2	59.8	< 0.01
Dialysate calcium (%)				
1.25 mmol/L	19.0	19.9	15.1	0.08
1.5 mmol/L	58.2	68.7	12.8	< 0.001
1.75 mmol/L	22.8	11.4	72.1	< 0.001
Elemental calcium (g/day)	1.07 ± 0.65	1.11 ± 0.67	0.91 ± 0.52	< 0.001
> 1.5 g/day (%)	28.4	30.4	19.5	< 0.01

Abbreviations: *HD*, haemodialysis; *PD*, peritoneal dialysis; *ESRD*, end-stage renal disease; *IQR*, interquartile range; SD, standard deviation.

Only 38.5%, 39.6% and 26.6% of participants were within the optimal level of serum P, corrected serum Ca and iPTH. There were no significant differences in percentages of achievement of optimal target in plasma P, Ca and iPTH between HD and PD patients. Serum P level and percentage of patients with hyperphosphatemia were statistically higher in the HD patients compared with those of PD patients, which was 6.3 ± 2.1 mg/dL vs 5.7 ± 2.0 mg/dL (P < 0.001) and 57.4% vs 47.4% (P < 0.01), respectively. Serum Ca level was higher in HD patients compared with PD patients, but no significant differences in percentages of patients with hypercalcemia or hypocalcemia. Serum iPTH level were statistically higher in the PD patients compared with those of HD patients (P = 0.03). And the percentage of patients with low PTH was lower in PD patients compared with HD patients (P < 0.001).

The percentages of patients with optimal P (37.6% vs 49.8% vs 54.5%, P < 0.01), Ca (38.6% vs 50.4% vs 56.0%, P < 0.01) and iPTH (26.5% vs 31.4% vs 32.1%, P < 0.01) were lower in HD patients, compared with those in the DOPPS 3 and DOPPS 4 (Table 2).

**Table 2:** Comparison of HD and DOPPS 3 and DOPPS 4 in mineral metabolism laboratory parameters

	**HD (n, %)**	**DOPPS 3 (n, %)**	**DOPPS 4 (n, %)**
Corrected calcium(mg/dL)			
<8.4	324 (19.4)*	791 (12.5)	904 (12.4)
8.4-9.5	647 (38.6)*	3195 (50.4)	4080 (56.0)
>9.5	703 (42.0)*	2355 (37.1)	2300 (31.6)
Phosphorus (mg/dL)			
<3.5	86 (5.0)*	765 (11.0)	895 (11.3)
3.5-5.5	642 (37.6)*	3476 (49.8)	4337 (54.5)
>5.5	981 (57.4)*	2738 (39.2)	2722 (34.2)
iPTH (pg/mL)			
<150	473 (29.0)*	2073 (36.8)	2325 (32.5)
150-300	431 (26.5)*	1767 (31.4)	2299 (32.1)
>300	724 (44.5)*	1787 (31.8)	2527 (35.3)

Abbreviations: *HD*, haemodialysis; *DOPPS*, Dialysis Outcomes and Practice Study.

*P<0.01 (HD group vs. DOPPS 3 and DOPPS 4).

DOPP 3(2007) and DOPP4 (2010): Includes data from AusNZ, Belgium, Canada, Germany, Italy, Japan, Spain, Sweden, UK, and US only.

Treatment regimen regarding MBD was different between HD and PD patients. More patients in HD were prescribed with phosphorus binders than those of PD patients (70.8% vs 64.0%,P = 0.03). More HD patients were prescribed with vitamin D than PD patients (69.2% vs 59.8%, P < 0.01). Compared with HD patients, the mean dose of elemental calcium intake was lower in PD patients (1.11 ± 0.67 g/day vs 0.91 ± 0.52 g/day, P < 0.01), and fewer patients exceeded the 1.5 g/day limit suggested by K/DOQI guidelines (30.4% vs 19.5%, P < 0.01). All phosphorus binders used in those patients were calcium-based binders. Among patients with serum phosphorus level above 5.5 mg/dL, 27.4% of HD patients and 25.0% of PD patients were not receiving any phosphorus binder. Actually in our study, 98% of patients who were prescribed vitamin D used calcitriol.

Serum P level and percentage of patients with hyperphosphatemia were statistically higher in the South region patients compared with those of North region patients, which was 6.7 ± 2.2 mg/dL vs 5.7 ± 1.8 mg/dL (P < 0.001) and 65.8% vs 45.6% (P < 0.01), respectively. To the best of our knowledge, the availability of phosphorus binders or vitamin D is not different in north and south China. And in our study, 70.5% patients in South region and 68.4% patients in North region were receiving phosphorus binder (*P* = 0.41). But more patients in North region were prescribed with vitamin D than those patients in South region (72.3% vs 62.7%, P = 0.045).

Table 3 presents the logistic regression analysis of hyperphosphatemia and various parameters. After adjusting for sex, serum albumin and hemoglobin, age, dialysis patterns and region of residency were still independently associated with hyperphosphatemia.

**Table 3:** The logistic regression analysis for hyperphosphatemia with different variables

**Variables**	**Crude OR (95% CI)**	Multivariable adjusted OR* (95% CI)
Age	0.99 (0.98-0.99)	0.99 (0.98-0.99)
Sex (female vs. male)	0.82 (0.69-0.99)	0.88 (0.73-1.07)
Dialysis patterns (PD vs. HD)	0.72 (0.57-0.92)	0.74 (0.57-0.95)
Serum albumin	1.00 (0.98-1.01)	1.00 (0.98-1.02)
Hemoglobin	1.00 (0.99-1.00)	1.00 (1.00-1.01)
region of residency (South vs. North)	2.14 (1.78-2.57)	2.15 (1.77-2.62)

OR* was adjusted for age, sex, dialytic patterns, serum albumin, hemoglobin, region of residency.

Abbreviations: *OR*, odds ration; *CI*, confidence interval.

## Discussion

In our cross-sectional survey involving various geographic regions in China, the treatment of MBD was found to be sub-optimal among patients receiving mountainous dialysis. Uncontrolled hyperphosphatemia is especially prominent.

Hyperphosphatemia is an independent risk factor for both mortality [1,10,11] and renal function decline in CKD patients [12,13]. A recent analysis of DOPPS [7] indicated that the lowest mortality was seen among patients with serum phosphorus 3.6 to 5.0 mg/dL, and higher mortality was observed in facilities with a higher proportion of patients with serum phosphorus greater than 6.0 mg/dL. Similarly, a meta-analysis indicated that the risk of death increased by 18% for every 1.0 mg/dL increase in serum phosphorus (relative risk of 1.18, 95% CI 1.12-1.25) in individuals with CKD [3]. Compared with results from DOPPS, control of hyperphosphatemia in our study is sub-optimal, which may be related to the following factors. Firstly, restriction of dietary phosphorus is the primary strategy for controlling hyperphosphatemia. Unfortunately, nutritionist was not available for most HD and PD centers in China. In our analysis, although we did not have information of dietary intake of phosphorus, residency in southern China was observed to be an independent risk factor for hyperphosphatemia. It is well known that in southern China, foods containing rich phosphorus (eg. seafood and meat soup) are more likely to be consumed than in Northern China. A study using 24-hour dietary recalls also indicated that phosphorus intake was higher among Southern Chinese compared with that of Northern Chinese, which was 562 ± 67 mg/1000 kcal and 378 ± 76 mg/1000 kcal, respectively [14]. Secondly, insufficient use of phosphorus binder was observed in our analysis. On the other hand, 46.5% of patients with serum calcium level above 9.5 mg/dL still were using calcium-containing phosphorus binder. In China, non-containing calcium phosphorus binders were not available. Therefore, inappropriate application of phosphorus binder may be related to hyperphosphatemia. Finally, our analysis revealed that HD was independently associated with higher risk of hyperphosphatemia, compared with PD patients. It has been shown that certain hemodialysis prescription may increase phosphorus clearance. Nocturnal HD [15,16] and short daily HD [17,18] was shown to reduce serum phosphorus levels and requirement for phosphorus binders. Membrane surface area itself has a potentially important impact on phosphorus removal. In a recent study in 18 patients over a period of 6 weeks, doubling of membrane area by the use of 2 dialyzers in parallel (with blood flow equally split between the dialyzers) resulted in a 1.34 mg/dL decline in predialysis serum phosphorus levels compared with conventional HD [19]. A recent randomized controlled trial evaluated the effect of different hemodialysis prescription on hyperphosphatemia among 493 patients [20]. It was shown that phosphorus levels decreased from 5.18 ± 0.10 mg/dL at baseline to 4.87 ± 0.10 mg/dL at 6 months in hemodiafiltration patients (P < 0.001) and were stable in low-flux HD patients (5.10 ± 0.10 mg/dL at baseline and 5.03 ± 0.10 mg/dL after 6 months, P = 0.5). After adjustment for phosphorus binder use, hemodiafiltration still have an advantage of improving phosphorus control over low-flux HD. In our study, low-flux HD was applied in the majority of our dialysis centers, which may also be related to poor phosphorus control. In summary, reducing phosphorus intake under the instruction of nutritionist, proper use of phosphorus binder, and adopting dialysis prescription with better phosphorus clearance may improve the sub-optimal treatment of hyperphosphatemia in China.

In K/DOQI guidelines, the optimal PTH levels was recommended to be 150-300 pg/ml for dialysis patients, which was based on the predictive ability of PTH for low-and high-turnover bone disease [8]. However, the early studies of MBD parameters evaluated survival as a function of a single time point baseline measurement of PTH, thereby ignoring important changes in the parameter [11]. Conversely, time dependent approaches are influenced strongly by events immediately proximal to the outcome and therefore may reflect worsening parameters caused by rapidly declining health status [10,21]. More recently, there have been attempts at using cumulative-effects models by the Hemodialysis (HEMO) Study investigators to better capture the relationship between bone mineral metabolism parameters and survival, and they also have found higher ceilings for PTH levels than previous work [22]. Analysis from the DOPPS also revealed an increased risk for all-cause mortality (not cardiovascular mortality) only when PTH > 600 pg/mL [7]. Therefore, in the recently released guidelines from the kidney Disease: Improving Global Outcomes (KDIGO), it is suggested that iPTH levels should be maintained in the range of approximately two to nine times the upper limit of normal for patients receiving dialysis [23]. In our study, about 56.7% of participants met the KDIGO target for PTH. However, more studies (especially interventional studies) are needed to verify the range suitable for better clinical outcome. In our study, the proportion of PD patients with PTH less than 150 pg/mL is not higher than HD patients. This may be related to the relatively low proportion of diabetes in PD patients and with short time on dialysis.

To date, there is no DOPPS-equivalent study looked at the management of mineral metabolism in PD patients. Compared with the data from Canadian PD patients [24], the present study demonstrated that the percentage of patients reached the K/DOQI targets for Ca (44.0% vs 44.7%) and iPTH (27.3% vs 28.4%) were similar among PD patients. However, the percentage of patients with hyperphosphatemia (> 5.5 mg/dL) and hypercalcemia (> 9.5 mg/dL) were higher than Canadian PD patients, which were 47.4% vs 21.2% and 36.6% vs 16.7%, respectively. It indicates that based on K/DOQI guidelines, Chinese PD patients are not within optimal target ranges and there is room for improvement.

Our study has several limitations that deserve mention. Firstly, study sites were enrolled on a voluntary basis, which might introduce bias. However, most of participants came from tertiary hospitals. Therefore, the direction of bias would be to overestimate the control rate of MBD. Secondly, some information of factors related to the MBD were not available in our study, such as diet, residual renal function, dialysis prescription and dialysis adequacy. Thirdly, laboratory tests were not performed in one central laboratory, therefore variability of measurement may affect the interpretation of our results. The same limitation exists for the comparison between our study and DOPPS study. Fourthly, the demographic characteristics and medical conditions of patients in our study might be different from those of participants in DOPPS study, which might contribute to the difference in parameters of MBD. Finally, the cross-sectional design limited our ability to make causal inference.

## Conclusion

Our national survey indicated that the status of MBD treatment is sub-optimal among Chinese patients receiving dialysis. The issue of hyperphosphatemia is prominent and needs to be improved. Multiple measures including dietary phosphorus restriction, oral phosphorus binders including non-containing calcium phosphorus binders and dialysis model should be optimized to improving the control of hyperphosphatemia, which in turn may reduce the risk for vascular calcification and cardiovascular mortality.

## Competing interests

The authors declare that they have no competing interests.

## Author’s contributions

K XL and Z LX participated in the study, analyzed the data, interpreted the results, and drafted the manuscript. Z L, C N, G Y, Y XQ, L WH, C JH, P LR, Y WJ, W H, C W, F MH, H LQ, D F, C XM, X ZY, Z JY, J Q, S W, X CY, T XL, H FF, S GY, M CL, W L, X DM and N ZH participated in the survey and study design and collected the data. Z L participated in the survey and study design and interpreted the results. W M formed the study concept, interpreted the results, and revised the manuscript. Wang HY revised the manuscript for important intellectual content. All authors read and approved the final manuscript.

## Pre-publication history

The pre-publication history for this paper can be accessed here:

http://www.biomedcentral.com/1471-2369/13/116/prepub
